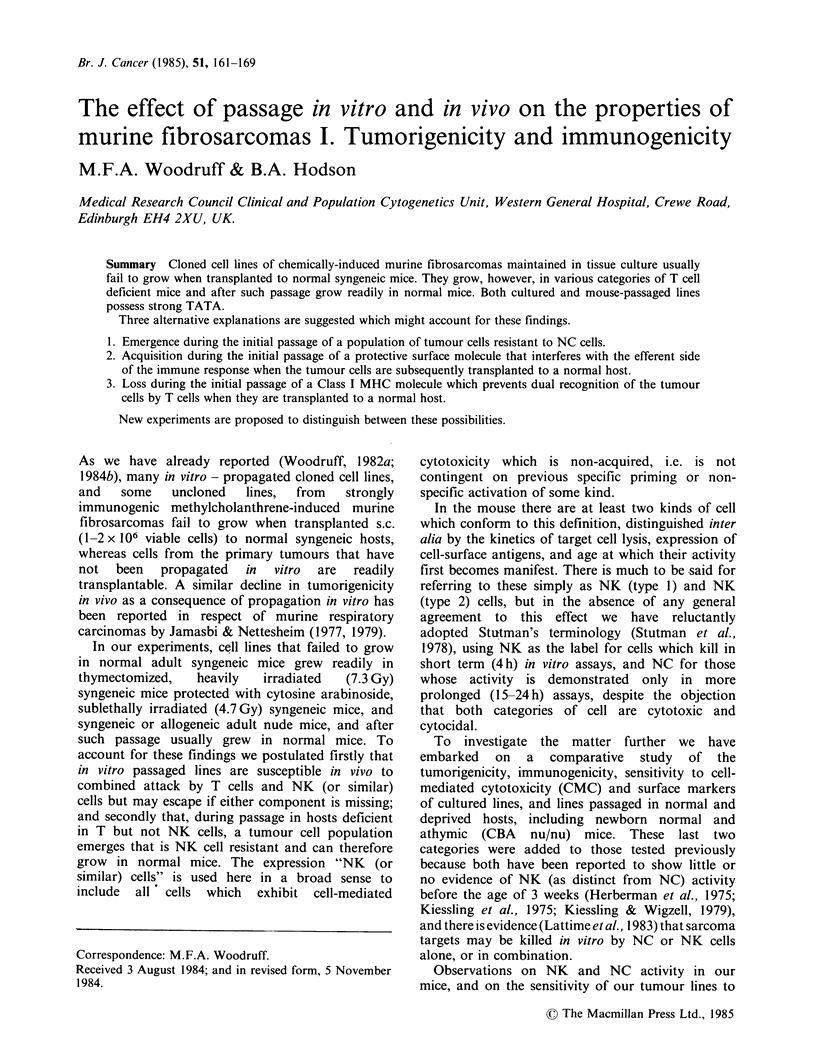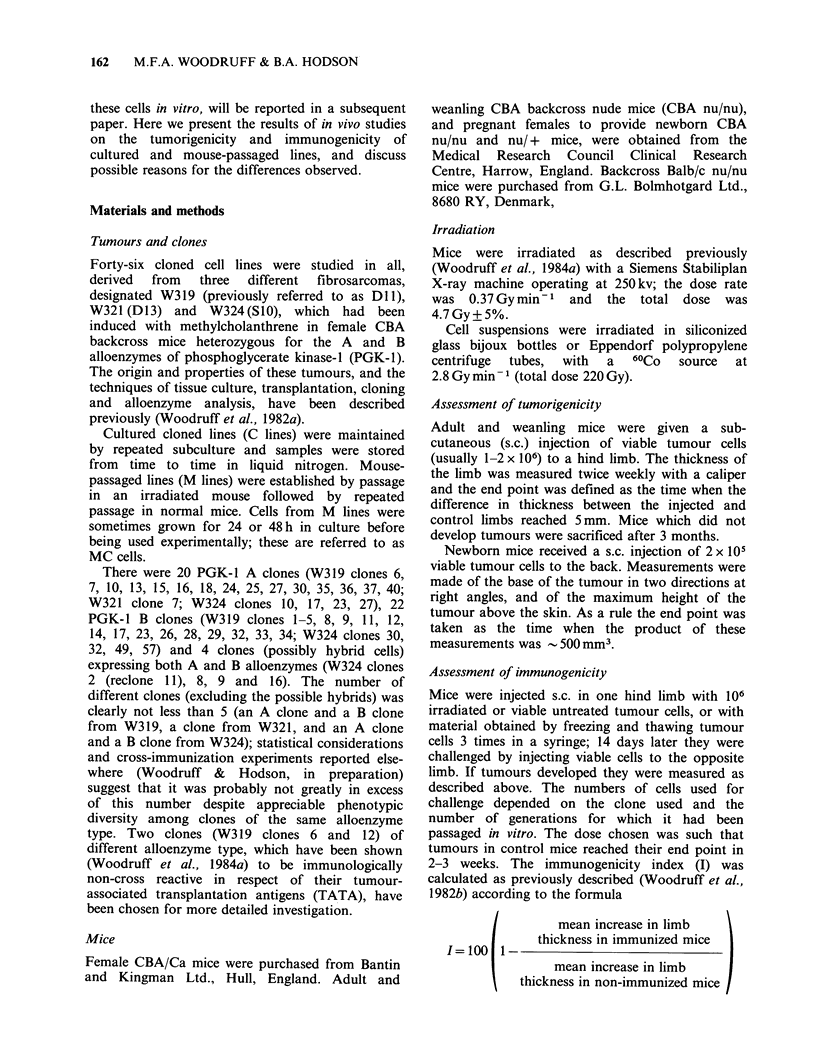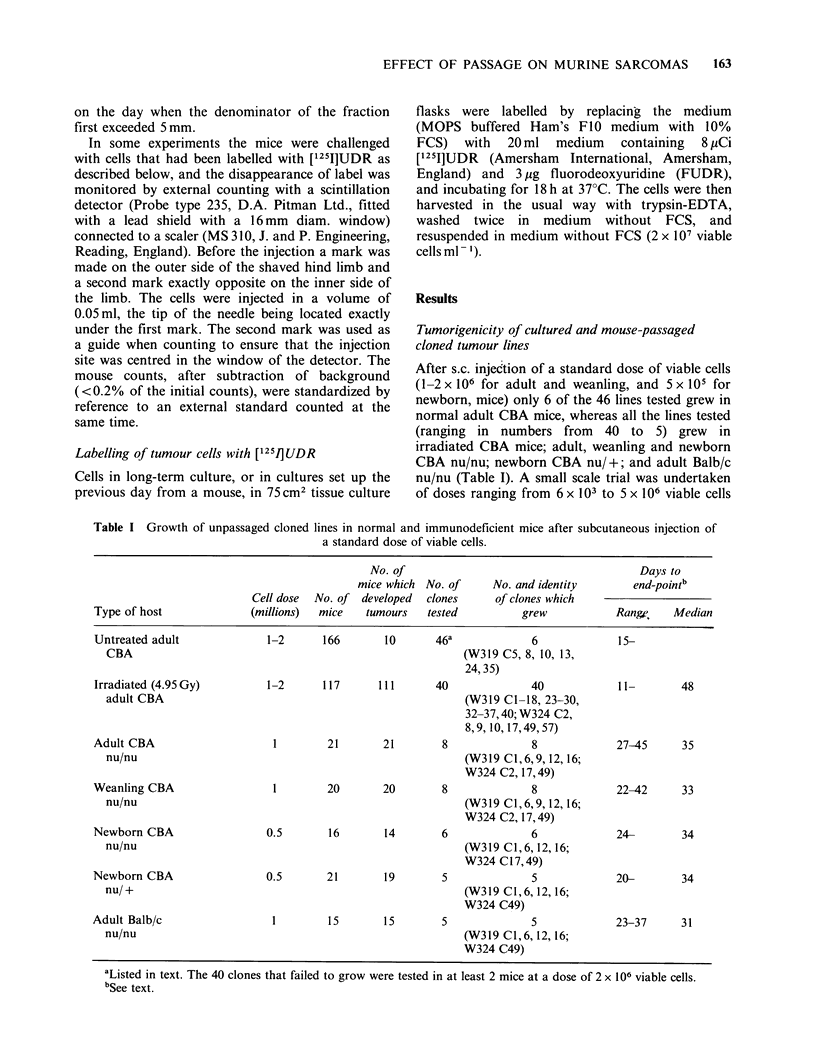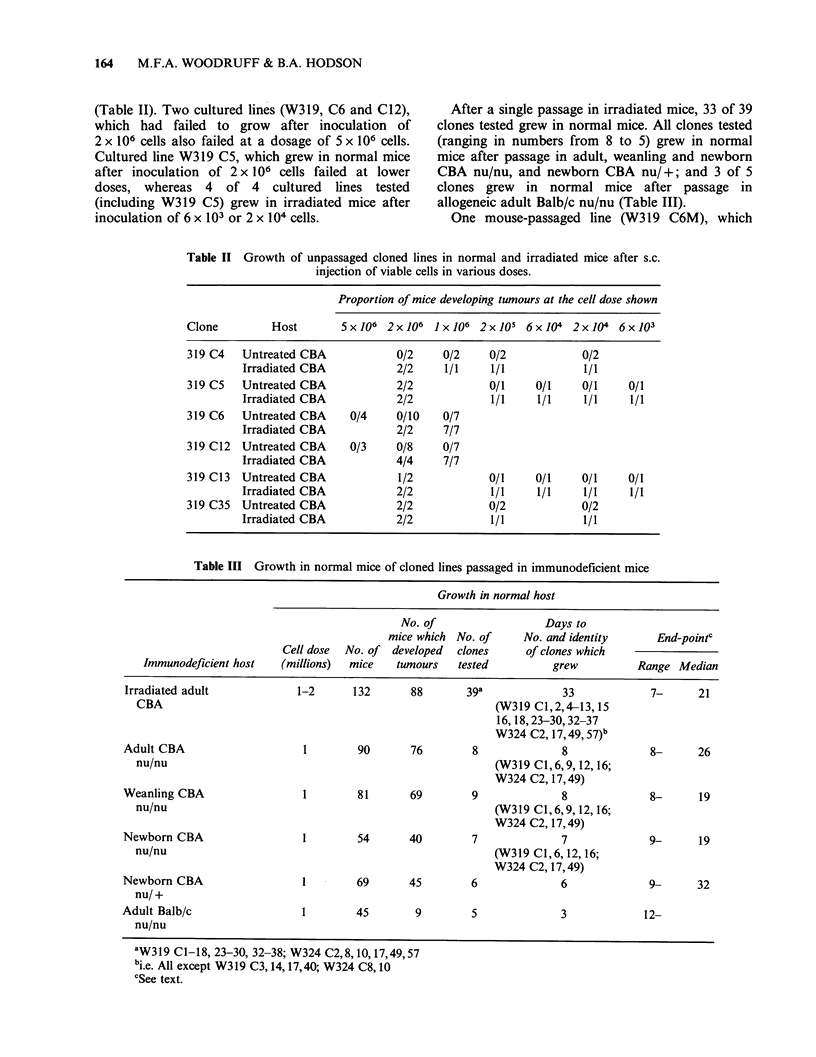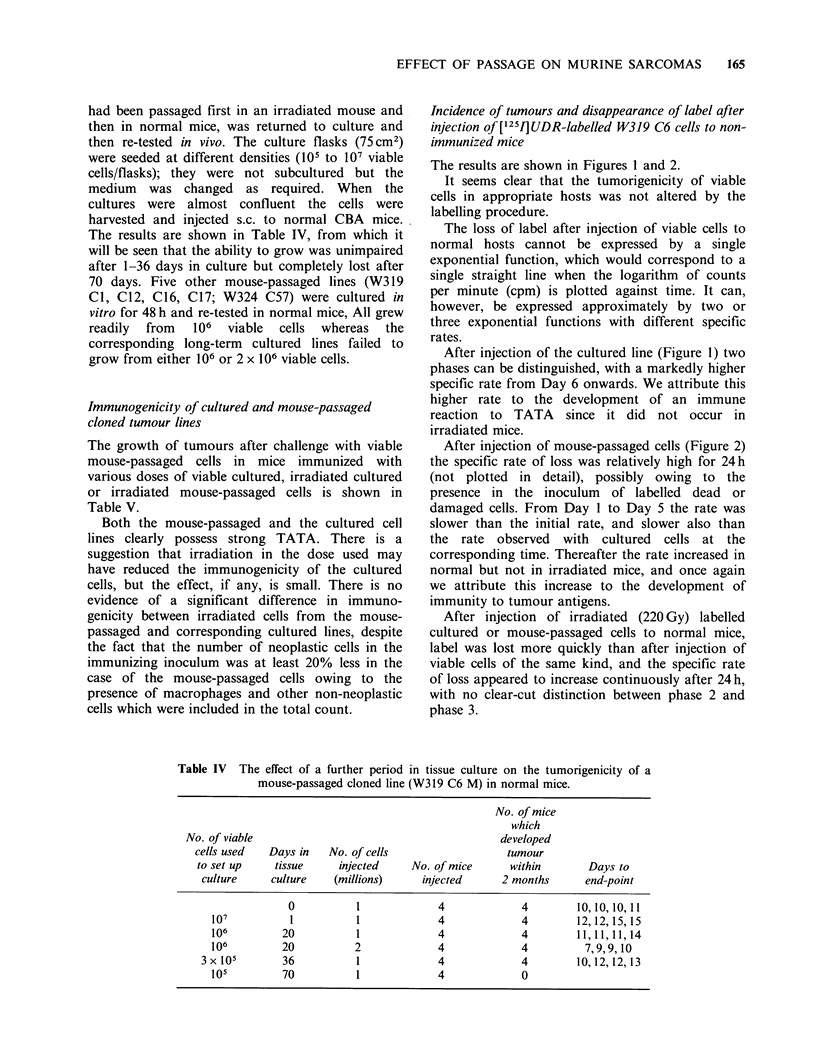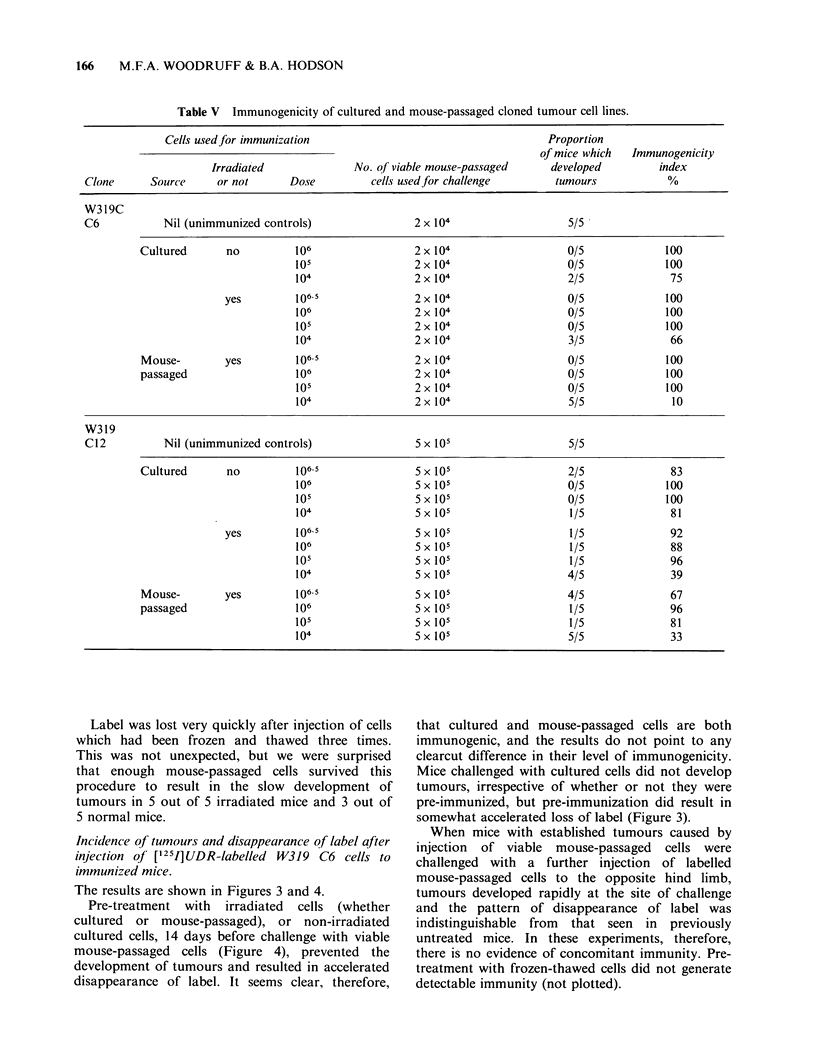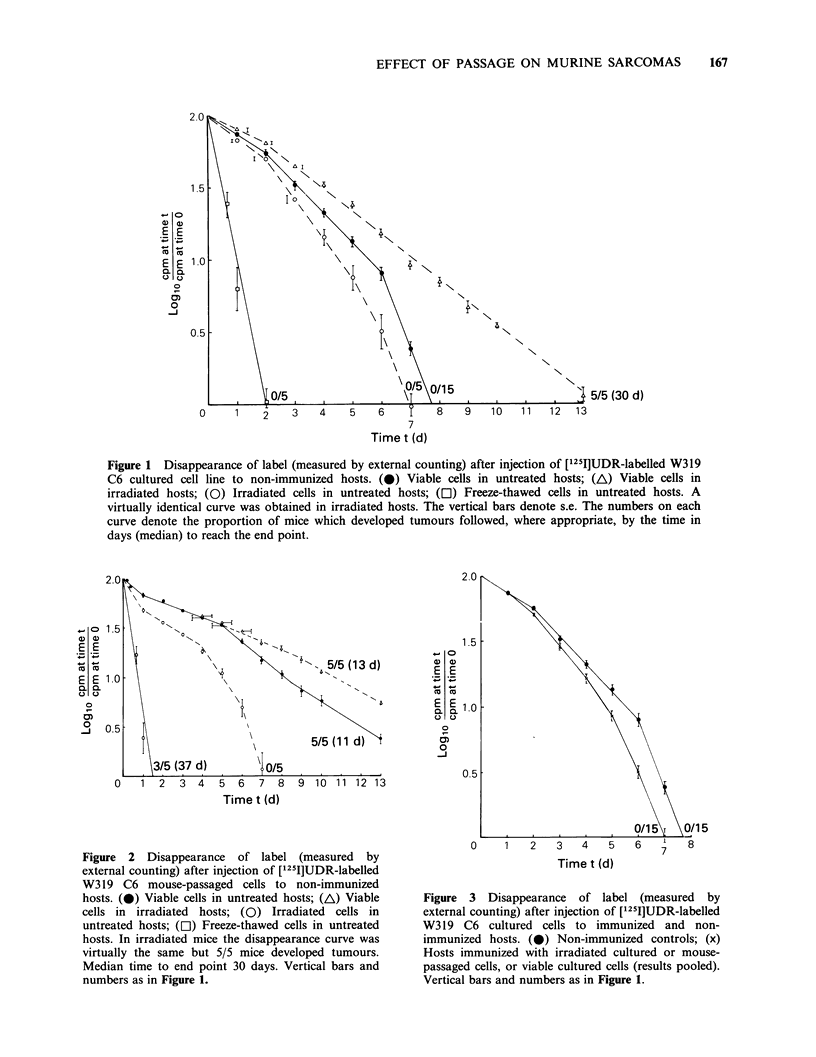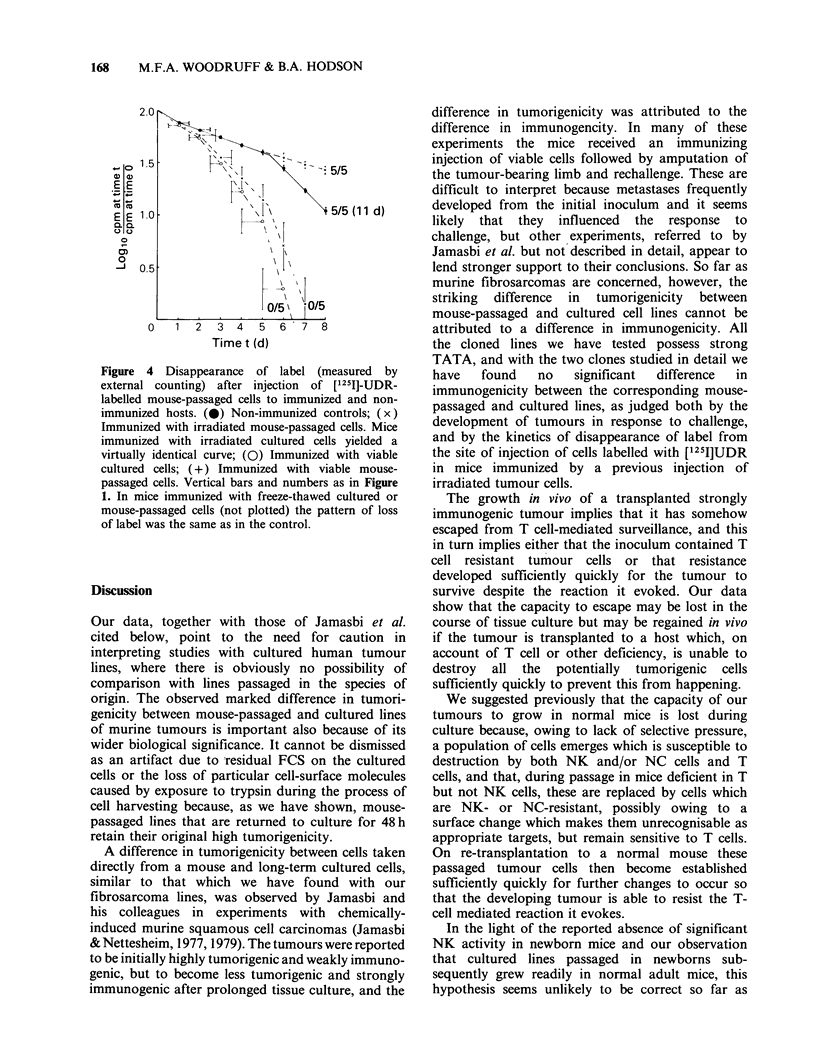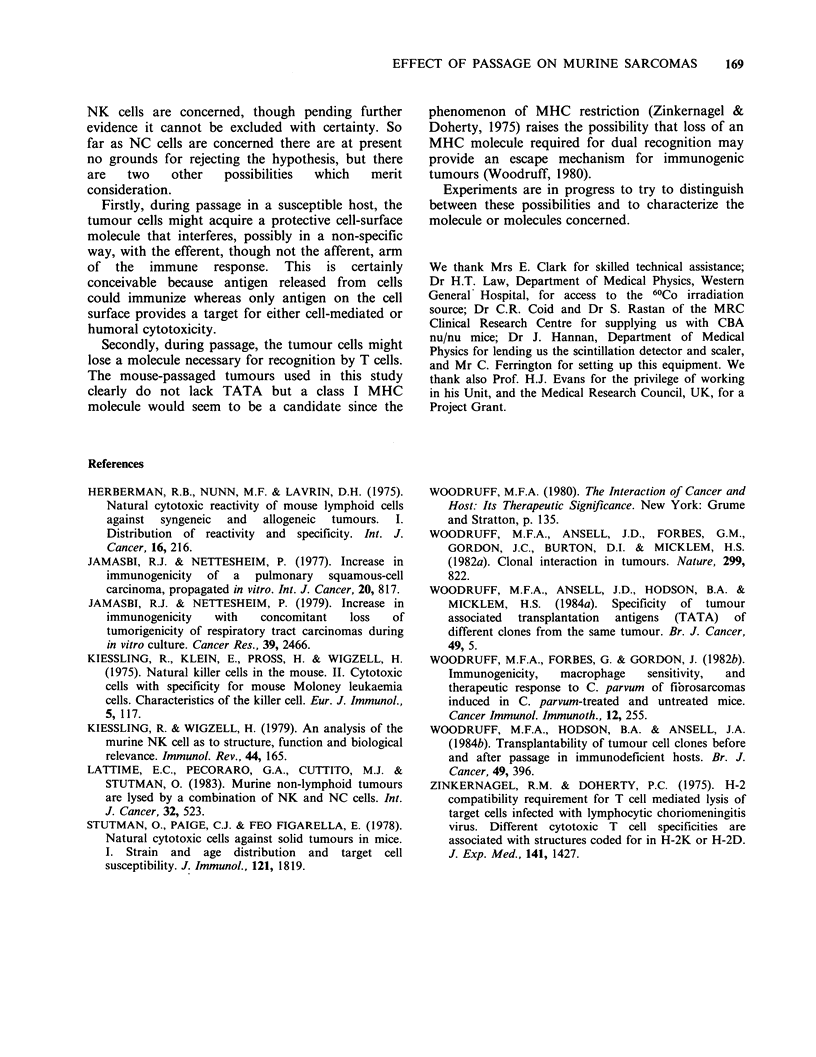# The effect of passage in vitro and in vivo on the properties of murine fibrosarcomas I. Tumorigenicity and immunogenicity.

**DOI:** 10.1038/bjc.1985.25

**Published:** 1985-02

**Authors:** M. F. Woodruff, B. A. Hodson

## Abstract

Cloned cell lines of chemically-induced murine fibrosarcomas maintained in tissue culture usually fail to grow when transplanted to normal syngeneic mice. They grow, however, in various categories of T cell deficient mice and after such passage grow readily in normal mice. Both cultured and mouse-passaged lines possess strong TATA. Three alternative explanations are suggested which might account for these findings. Emergence during the initial passage of a population of tumour cells resistant to NC cells. Acquisition during the initial passage of a protective surface molecule that interferes with the efferent side of the immune response when the tumour cells are subsequently transplanted to a normal host. Loss during the initial passage of a Class I MHC molecule which prevents dual recognition of the tumour cells by T cells when they are transplanted to a normal host. New experiments are proposed to distinguish between these possibilities.


*Br. J. Cancer* (1985), **51**, 161–169

### The effect of passage *in vitro* and *in vivo* on the properties of murine fibrosarcomas I. Tumorigenicity and immunogenicity

M.F.A. Woodruff & B.A. Hodson

*Medical Research Council Clinical and Population Cytogenetics Unit, Western General Hospital, Crewe Road, Edinburgh EH4 2XU, UK.*

**Summary** Cloned cell lines of chemically-induced murine fibrosarcomas maintained in tissue culture usually fail to grow when transplanted to normal syngeneic mice. They grow, however, in various categories of T cell deficient mice and after such passage grow readily in normal mice. Both cultured and mouse-passaged lines possess strong TATA.

Three alternative explanations are suggested which might account for these findings.

1. Emergence during the initial passage of a population of tumour cells resistant to NC cells.
2. Acquisition during the initial passage of a protective surface molecule that interferes with the efferent side of the immune response when the tumour cells are subsequently transplanted to a normal host.
3. Loss during the initial passage of a Class I MHC molecule which prevents dual recognition of the tumour cells by T cells when they are transplanted to a normal host.

New experiments are proposed to distinguish between these possibilities.

As we have already reported (Woodruff, 1982*a*; 1984*b*), many *in vitro* – propagated cloned cell lines, and some uncloned lines, from strongly immunogenic methylcholanthrene-induced murine fibrosarcomas fail to grow when transplanted s.c. ($1–2 \times 10^6$ viable cells) to normal syngeneic hosts, whereas cells from the primary tumours that have not been propagated *in vitro* are readily transplantable. A similar decline in tumorigenicity *in vivo* as a consequence of propagation *in vitro* has been reported in respect of murine respiratory carcinomas by Jamasbi & Nettesheim (1977, 1979).

In our experiments, cell lines that failed to grow in normal adult syngeneic mice grew readily in thymectomized, heavily irradiated (7.3 Gy) syngeneic mice protected with cytosine arabinoside, sublethally irradiated (4.7 Gy) syngeneic mice, and syngeneic or allogeneic adult nude mice, and after such passage usually grew in normal mice. To account for these findings we postulated firstly that *in vitro* passaged lines are susceptible *in vivo* to combined attack by T cells and NK (or similar) cells but may escape if either component is missing; and secondly that, during passage in hosts deficient in T but not NK cells, a tumour cell population emerges that is NK cell resistant and can therefore grow in normal mice. The expression "NK (or similar) cells" is used here in a broad sense to include all cells which exhibit cell-mediated cytotoxicity which is non-acquired, i.e. is not contingent on previous specific priming or non-specific activation of some kind.

In the mouse there are at least two kinds of cell which conform to this definition, distinguished *inter alia* by the kinetics of target cell lysis, expression of cell-surface antigens, and age at which their activity first becomes manifest. There is much to be said for referring to these simply as NK (type 1) and NK (type 2) cells, but in the absence of any general agreement to this effect we have reluctantly adopted Stutman's terminology (Stutman *et al.*, 1978), using NK as the label for cells which kill in short term (4 h) *in vitro* assays, and NC for those whose activity is demonstrated only in more prolonged (15–24 h) assays, despite the objection that both categories of cell are cytotoxic and cytocidal.

To investigate the matter further we have embarked on a comparative study of the tumorigenicity, immunogenicity, sensitivity to cell-mediated cytotoxicity (CMC) and surface markers of cultured lines, and lines passaged in normal and deprived hosts, including newborn normal and athymic (CBA nu/nu) mice. These last two categories were added to those tested previously because both have been reported to show little or no evidence of NK (as distinct from NC) activity before the age of 3 weeks (Herberman *et al.*, 1975; Kiessling *et al.*, 1975; Kiessling & Wigzell, 1979), and there is evidence (Lattime *et al.*, 1983) that sarcoma targets may be killed *in vitro* by NC or NK cells alone, or in combination.

Observations on NK and NC activity in our mice, and on the sensitivity of our tumour lines to

Correspondence: M.F.A. Woodruff.

Received 3 August 1984; and in revised form, 5 November 1984.

© The Macmillan Press Ltd., 1985

these cells *in vitro,* will be reported in a subsequent paper. Here we present the results of *in vivo* studies on the tumorigenicity and immunogenicity of cultured and mouse-passaged lines, and discuss possible reasons for the differences observed.

### Materials and methods

### *Tumours and clones*

Forty-six cloned cell lines were studied in all, derived from three different fibrosarcomas, designated W319 (previously referred to as D11), W321 (D13) and W324 (S10), which had been induced with methylcholanthrene in female CBA backcross mice heterozygous for the A and B alloenzymes of phosphoglycerate kinase-1 (PGK-1). The origin and properties of these tumours, and the techniques of tissue culture, transplantation, cloning and alloenzyme analysis, have been described previously (Woodruff *et al.*, 1982*a*).

Cultured cloned lines (C lines) were maintained by repeated subculture and samples were stored from time to time in liquid nitrogen. Mouse-passaged lines (M lines) were established by passage in an irradiated mouse followed by repeated passage in normal mice. Cells from M lines were sometimes grown for 24 or 48 h in culture before being used experimentally; these are referred to as MC cells.

There were 20 PGK-1 A clones (W319 clones 6, 7, 10, 13, 15, 16, 18, 24, 25, 27, 30, 35, 36, 37, 40; W321 clone 7; W324 clones 10, 17, 23, 27), 22 PGK-1 B clones (W319 clones 1-5, 8, 9, 11, 12, 14, 17, 23, 26, 28, 29, 32, 33, 34; W324 clones 30, 32, 49, 57) and 4 clones (possibly hybrid cells) expressing both A and B alloenzymes (W324 clones 2 (reclone 11), 8, 9 and 16). The number of different clones (excluding the possible hybrids) was clearly not less than 5 (an A clone and a B clone from W319, a clone from W321, and an A clone and a B clone from W324); statistical considerations and cross-immunization experiments reported elsewhere (Woodruff & Hodson, in preparation) suggest that it was probably not greatly in excess of this number despite appreciable phenotypic diversity among clones of the same alloenzyme type. Two clones (W319 clones 6 and 12) of different alloenzyme type, which have been shown (Woodruff *et al.*, 1984*a*) to be immunologically non-cross reactive in respect of their tumour-associated transplantation antigens (TATA), have been chosen for more detailed investigation.

### *Mice*

Female CBA/Ca mice were purchased from Bantin and Kingman Ltd., Hull, England. Adult and weanling CBA backcross nude mice (CBA nu/nu), and pregnant females to provide newborn CBA nu/nu and nu/+ mice, were obtained from the Medical Research Council Clinical Research Centre, Harrow, England. Backcross Balb/c nu/nu mice were purchased from G.L. Bolmhotgard Ltd., 8680 RY, Denmark,

### *Irradiation*

Mice were irradiated as described previously (Woodruff *et al.*, 1984*a*) with a Siemens Stabiliplan X-ray machine operating at 250 kv; the dose rate was $0.37\,\mathrm{Gy\,min^{-1}}$ and the total dose was $4.7\,\mathrm{Gy} \pm 5\%$.

Cell suspensions were irradiated in siliconized glass bijoux bottles or Eppendorf polypropylene centrifuge tubes, with a $^{60}$Co source at $2.8\,\mathrm{Gy\,min^{-1}}$ (total dose 220 Gy).

### *Assessment of tumorigenicity*

Adult and weanling mice were given a subcutaneous (s.c.) injection of viable tumour cells (usually $1\text{–}2 \times 10^6$) to a hind limb. The thickness of the limb was measured twice weekly with a caliper and the end point was defined as the time when the difference in thickness between the injected and control limbs reached 5 mm. Mice which did not develop tumours were sacrificed after 3 months.

Newborn mice received a s.c. injection of $2 \times 10^5$ viable tumour cells to the back. Measurements were made of the base of the tumour in two directions at right angles, and of the maximum height of the tumour above the skin. As a rule the end point was taken as the time when the product of these measurements was $\sim 500\,\mathrm{mm}^3$.

### *Assessment of immunogenicity*

Mice were injected s.c. in one hind limb with $10^6$ irradiated or viable untreated tumour cells, or with material obtained by freezing and thawing tumour cells 3 times in a syringe; 14 days later they were challenged by injecting viable cells to the opposite limb. If tumours developed they were measured as described above. The numbers of cells used for challenge depended on the clone used and the number of generations for which it had been passaged *in vitro*. The dose chosen was such that tumours in control mice reached their end point in 2–3 weeks. The immunogenicity index (I) was calculated as previously described (Woodruff *et al.*, 1982*b*) according to the formula

$$I = 100\left(1 - \frac{\text{mean increase in limb thickness in immunized mice}}{\text{mean increase in limb thickness in non-immunized mice}}\right)$$

on the day when the denominator of the fraction first exceeded 5 mm.

In some experiments the mice were challenged with cells that had been labelled with [$^{125}$I]UDR as described below, and the disappearance of label was monitored by external counting with a scintillation detector (Probe type 235, D.A. Pitman Ltd., fitted with a lead shield with a 16 mm diam. window) connected to a scaler (MS 310, J. and P. Engineering, Reading, England). Before the injection a mark was made on the outer side of the shaved hind limb and a second mark exactly opposite on the inner side of the limb. The cells were injected in a volume of 0.05 ml, the tip of the needle being located exactly under the first mark. The second mark was used as a guide when counting to ensure that the injection site was centred in the window of the detector. The mouse counts, after subtraction of background ($<0.2\%$ of the initial counts), were standardized by reference to an external standard counted at the same time.

### *Labelling of tumour cells with [$^{125}$I]UDR*

Cells in long-term culture, or in cultures set up the previous day from a mouse, in 75 cm$^2$ tissue culture flasks were labelled by replacing the medium (MOPS buffered Ham's F10 medium with 10% FCS) with 20 ml medium containing 8 $\mu$Ci [$^{125}$I]UDR (Amersham International, Amersham, England) and 3 $\mu$g fluorodeoxyuridine (FUDR), and incubating for 18 h at 37°C. The cells were then harvested in the usual way with trypsin-EDTA, washed twice in medium without FCS, and resuspended in medium without FCS ($2 \times 10^7$ viable cells ml$^{-1}$).

### Results

### *Tumorigenicity of cultured and mouse-passaged cloned tumour lines*

After s.c. injection of a standard dose of viable cells ($1\text{–}2 \times 10^6$ for adult and weanling, and $5 \times 10^5$ for newborn, mice) only 6 of the 46 lines tested grew in normal adult CBA mice, whereas all the lines tested (ranging in numbers from 40 to 5) grew in irradiated CBA mice; adult, weanling and newborn CBA nu/nu; newborn CBA nu/+; and adult Balb/c nu/nu (Table I). A small scale trial was undertaken of doses ranging from $6 \times 10^3$ to $5 \times 10^6$ viable cells

**Table I** Growth of unpassaged cloned lines in normal and immunodeficient mice after subcutaneous injection of a standard dose of viable cells.

| Type of host | *Cell dose (millions)* | *No. of mice* | *No. of mice which developed tumours* | *No. of clones tested* | *No. and identity of clones which grew* | *Days to end-point*[b] | |
|---|---|---|---|---|---|---|---|
| | | | | | | *Range* | *Median* |
| Untreated adult CBA | 1–2 | 166 | 10 | 46[a] | 6 (W319 C5, 8, 10, 13, 24, 35) | 15– | |
| Irradiated (4.95 Gy) adult CBA | 1–2 | 117 | 111 | 40 | 40 (W319 C1–18, 23–30, 32–37, 40; W324 C2, 8, 9, 10, 17, 49, 57) | 11– | 48 |
| Adult CBA nu/nu | 1 | 21 | 21 | 8 | 8 (W319 C1, 6, 9, 12, 16; W324 C2, 17, 49) | 27–45 | 35 |
| Weanling CBA nu/nu | 1 | 20 | 20 | 8 | 8 (W319 C1, 6, 9, 12, 16; W324 C2, 17, 49) | 22–42 | 33 |
| Newborn CBA nu/nu | 0.5 | 16 | 14 | 6 | 6 (W319 C1, 6, 12, 16; W324 C17, 49) | 24– | 34 |
| Newborn CBA nu/+ | 0.5 | 21 | 19 | 5 | 5 (W319 C1, 6, 12, 16; W324 C49) | 20– | 34 |
| Adult Balb/c nu/nu | 1 | 15 | 15 | 5 | 5 (W319 C1, 6, 12, 16; W324 C49) | 23–37 | 31 |

[a]Listed in text. The 40 clones that failed to grow were tested in at least 2 mice at a dose of $2 \times 10^6$ viable cells.
[b]See text.

(Table II). Two cultured lines (W319, C6 and C12), which had failed to grow after inoculation of $2 \times 10^6$ cells also failed at a dosage of $5 \times 10^6$ cells. Cultured line W319 C5, which grew in normal mice after inoculation of $2 \times 10^6$ cells failed at lower doses, whereas 4 of 4 cultured lines tested (including W319 C5) grew in irradiated mice after inoculation of $6 \times 10^3$ or $2 \times 10^4$ cells.

After a single passage in irradiated mice, 33 of 39 clones tested grew in normal mice. All clones tested (ranging in numbers from 8 to 5) grew in normal mice after passage in adult, weanling and newborn CBA nu/nu, and newborn CBA nu/+; and 3 of 5 clones grew in normal mice after passage in allogeneic adult Balb/c nu/nu (Table III).

One mouse-passaged line (W319 C6M), which

**Table II** Growth of unpassaged cloned lines in normal and irradiated mice after s.c. injection of viable cells in various doses.

| | | *Proportion of mice developing tumours at the cell dose shown* | | | | | | |
|---|---|---|---|---|---|---|---|---|
| Clone | Host | $5 \times 10^6$ | $2 \times 10^6$ | $1 \times 10^6$ | $2 \times 10^5$ | $6 \times 10^4$ | $2 \times 10^4$ | $6 \times 10^3$ |
| 319 C4 | Untreated CBA | | 0/2 | 0/2 | 0/2 | | 0/2 | |
| | Irradiated CBA | | 2/2 | 1/1 | 1/1 | | 1/1 | |
| 319 C5 | Untreated CBA | | 2/2 | | 0/1 | 0/1 | 0/1 | 0/1 |
| | Irradiated CBA | | 2/2 | | 1/1 | 1/1 | 1/1 | 1/1 |
| 319 C6 | Untreated CBA | 0/4 | 0/10 | 0/7 | | | | |
| | Irradiated CBA | | 2/2 | 7/7 | | | | |
| 319 C12 | Untreated CBA | 0/3 | 0/8 | 0/7 | | | | |
| | Irradiated CBA | | 4/4 | 7/7 | | | | |
| 319 C13 | Untreated CBA | | 1/2 | | 0/1 | 0/1 | 0/1 | 0/1 |
| | Irradiated CBA | | 2/2 | | 1/1 | 1/1 | 1/1 | 1/1 |
| 319 C35 | Untreated CBA | | 2/2 | | 0/2 | | 0/2 | |
| | Irradiated CBA | | 2/2 | | 1/1 | | 1/1 | |

**Table III** Growth in normal mice of cloned lines passaged in immunodeficient mice

| | *Growth in normal host* | | | | | | |
|---|---|---|---|---|---|---|---|
| | | | | | | *End-point*[c] | |
| *Immunodeficient host* | *Cell dose (millions)* | *No. of mice* | *No. of mice which developed tumours* | *No. of clones tested* | *Days to No. and identity of clones which grew* | *Range* | *Median* |
| Irradiated adult CBA | 1–2 | 132 | 88 | 39[a] | 33 (W319 C1, 2, 4–13, 15 16, 18, 23–30, 32–37 W324 C2, 17, 49, 57)[b] | 7– | 21 |
| Adult CBA nu/nu | 1 | 90 | 76 | 8 | 8 (W319 C1, 6, 9, 12, 16; W324 C2, 17, 49) | 8– | 26 |
| Weanling CBA nu/nu | 1 | 81 | 69 | 9 | 8 (W319 C1, 6, 9, 12, 16; W324 C2, 17, 49) | 8– | 19 |
| Newborn CBA nu/nu | 1 | 54 | 40 | 7 | 7 (W319 C1, 6, 12, 16; W324 C2, 17, 49) | 9– | 19 |
| Newborn CBA nu/+ | 1 | 69 | 45 | 6 | 6 | 9– | 32 |
| Adult Balb/c nu/nu | 1 | 45 | 9 | 5 | 3 | 12– | |

[a]W319 C1–18, 23–30, 32–38; W324 C2, 8, 10, 17, 49, 57
[b]i.e. All except W319 C3, 14, 17, 40; W324 C8, 10
[c]See text.

had been passaged first in an irradiated mouse and then in normal mice, was returned to culture and then re-tested *in vivo*. The culture flasks (75 $cm^2$) were seeded at different densities ($10^5$ to $10^7$ viable cells/flasks); they were not subcultured but the medium was changed as required. When the cultures were almost confluent the cells were harvested and injected s.c. to normal CBA mice. The results are shown in Table IV, from which it will be seen that the ability to grow was unimpaired after 1–36 days in culture but completely lost after 70 days. Five other mouse-passaged lines (W319 C1, C12, C16, C17; W324 C57) were cultured *in vitro* for 48 h and re-tested in normal mice, All grew readily from $10^6$ viable cells whereas the corresponding long-term cultured lines failed to grow from either $10^6$ or $2 \times 10^6$ viable cells.

*Immunogenicity of cultured and mouse-passaged cloned tumour lines*

The growth of tumours after challenge with viable mouse-passaged cells in mice immunized with various doses of viable cultured, irradiated cultured or irradiated mouse-passaged cells is shown in Table V.

Both the mouse-passaged and the cultured cell lines clearly possess strong TATA. There is a suggestion that irradiation in the dose used may have reduced the immunogenicity of the cultured cells, but the effect, if any, is small. There is no evidence of a significant difference in immunogenicity between irradiated cells from the mouse-passaged and corresponding cultured lines, despite the fact that the number of neoplastic cells in the immunizing inoculum was at least 20% less in the case of the mouse-passaged cells owing to the presence of macrophages and other non-neoplastic cells which were included in the total count.

*Incidence of tumours and disappearance of label after injection of [$^{125}I$]UDR-labelled W319 C6 cells to non-immunized mice*

The results are shown in Figures 1 and 2.

It seems clear that the tumorigenicity of viable cells in appropriate hosts was not altered by the labelling procedure.

The loss of label after injection of viable cells to normal hosts cannot be expressed by a single exponential function, which would correspond to a single straight line when the logarithm of counts per minute (cpm) is plotted against time. It can, however, be expressed approximately by two or three exponential functions with different specific rates.

After injection of the cultured line (Figure 1) two phases can be distinguished, with a markedly higher specific rate from Day 6 onwards. We attribute this higher rate to the development of an immune reaction to TATA since it did not occur in irradiated mice.

After injection of mouse-passaged cells (Figure 2) the specific rate of loss was relatively high for 24 h (not plotted in detail), possibly owing to the presence in the inoculum of labelled dead or damaged cells. From Day 1 to Day 5 the rate was slower than the initial rate, and slower also than the rate observed with cultured cells at the corresponding time. Thereafter the rate increased in normal but not in irradiated mice, and once again we attribute this increase to the development of immunity to tumour antigens.

After injection of irradiated (220 Gy) labelled cultured or mouse-passaged cells to normal mice, label was lost more quickly than after injection of viable cells of the same kind, and the specific rate of loss appeared to increase continuously after 24 h, with no clear-cut distinction between phase 2 and phase 3.

**Table IV** The effect of a further period in tissue culture on the tumorigenicity of a mouse-passaged cloned line (W319 C6 M) in normal mice.

| *No. of viable cells used to set up culture* | *Days in tissue culture* | *No. of cells injected (millions)* | *No. of mice injected* | *No. of mice which developed tumour within 2 months* | *Days to end-point* |
|---|---|---|---|---|---|
| | 0 | 1 | 4 | 4 | 10, 10, 10, 11 |
| $10^7$ | 1 | 1 | 4 | 4 | 12, 12, 15, 15 |
| $10^6$ | 20 | 1 | 4 | 4 | 11, 11, 11, 14 |
| $10^6$ | 20 | 2 | 4 | 4 | 7, 9, 9, 10 |
| $3 \times 10^5$ | 36 | 1 | 4 | 4 | 10, 12, 12, 13 |
| $10^5$ | 70 | 1 | 4 | 0 | |

**Table V** Immunogenicity of cultured and mouse-passaged cloned tumour cell lines.

| *Clone* | *Cells used for immunization* *Source* | *Irradiated or not* | *Dose* | *No. of viable mouse-passaged cells used for challenge* | *Proportion of mice which developed tumours* | *Immunogenicity index %* |
|---|---|---|---|---|---|---|
| W319C C6 | Nil (unimmunized controls) | | | $2\times10^4$ | 5/5 | |
| | Cultured | no | $10^6$ | $2\times10^4$ | 0/5 | 100 |
| | | | $10^5$ | $2\times10^4$ | 0/5 | 100 |
| | | | $10^4$ | $2\times10^4$ | 2/5 | 75 |
| | | yes | $10^{6.5}$ | $2\times10^4$ | 0/5 | 100 |
| | | | $10^6$ | $2\times10^4$ | 0/5 | 100 |
| | | | $10^5$ | $2\times10^4$ | 0/5 | 100 |
| | | | $10^4$ | $2\times10^4$ | 3/5 | 66 |
| | Mouse-passaged | yes | $10^{6.5}$ | $2\times10^4$ | 0/5 | 100 |
| | | | $10^6$ | $2\times10^4$ | 0/5 | 100 |
| | | | $10^5$ | $2\times10^4$ | 0/5 | 100 |
| | | | $10^4$ | $2\times10^4$ | 5/5 | 10 |
| W319 C12 | Nil (unimmunized controls) | | | $5\times10^5$ | 5/5 | |
| | Cultured | no | $10^{6.5}$ | $5\times10^5$ | 2/5 | 83 |
| | | | $10^6$ | $5\times10^5$ | 0/5 | 100 |
| | | | $10^5$ | $5\times10^5$ | 0/5 | 100 |
| | | | $10^4$ | $5\times10^5$ | 1/5 | 81 |
| | | yes | $10^{6.5}$ | $5\times10^5$ | 1/5 | 92 |
| | | | $10^6$ | $5\times10^5$ | 1/5 | 88 |
| | | | $10^5$ | $5\times10^5$ | 1/5 | 96 |
| | | | $10^4$ | $5\times10^5$ | 4/5 | 39 |
| | Mouse-passaged | yes | $10^{6.5}$ | $5\times10^5$ | 4/5 | 67 |
| | | | $10^6$ | $5\times10^5$ | 1/5 | 96 |
| | | | $10^5$ | $5\times10^5$ | 1/5 | 81 |
| | | | $10^4$ | $5\times10^5$ | 5/5 | 33 |

Label was lost very quickly after injection of cells which had been frozen and thawed three times. This was not unexpected, but we were surprised that enough mouse-passaged cells survived this procedure to result in the slow development of tumours in 5 out of 5 irradiated mice and 3 out of 5 normal mice.

*Incidence of tumours and disappearance of label after injection of* [$^{125}$*I*]*UDR-labelled W319 C6 cells to immunized mice.*

The results are shown in Figures 3 and 4.

Pre-treatment with irradiated cells (whether cultured or mouse-passaged), or non-irradiated cultured cells, 14 days before challenge with viable mouse-passaged cells (Figure 4), prevented the development of tumours and resulted in accelerated disappearance of label. It seems clear, therefore, that cultured and mouse-passaged cells are both immunogenic, and the results do not point to any clearcut difference in their level of immunogenicity. Mice challenged with cultured cells did not develop tumours, irrespective of whether or not they were pre-immunized, but pre-immunization did result in somewhat accelerated loss of label (Figure 3).

When mice with established tumours caused by injection of viable mouse-passaged cells were challenged with a further injection of labelled mouse-passaged cells to the opposite hind limb, tumours developed rapidly at the site of challenge and the pattern of disappearance of label was indistinguishable from that seen in previously untreated mice. In these experiments, therefore, there is no evidence of concomitant immunity. Pre-treatment with frozen-thawed cells did not generate detectable immunity (not plotted).

**Figure 1** Disappearance of label (measured by external counting) after injection of [$^{125}$I]UDR-labelled W319 C6 cultured cell line to non-immunized hosts. (●) Viable cells in untreated hosts; (△) Viable cells in irradiated hosts; (○) Irradiated cells in untreated hosts; (□) Freeze-thawed cells in untreated hosts. A virtually identical curve was obtained in irradiated hosts. The vertical bars denote s.e. The numbers on each curve denote the proportion of mice which developed tumours followed, where appropriate, by the time in days (median) to reach the end point.

**Figure 2** Disappearance of label (measured by external counting) after injection of [$^{125}$I]UDR-labelled W319 C6 mouse-passaged cells to non-immunized hosts. (●) Viable cells in untreated hosts; (△) Viable cells in irradiated hosts; (○) Irradiated cells in untreated hosts; (□) Freeze-thawed cells in untreated hosts. In irradiated mice the disappearance curve was virtually the same but 5/5 mice developed tumours. Median time to end point 30 days. Vertical bars and numbers as in **Figure 1.**

**Figure 3** Disappearance of label (measured by external counting) after injection of [$^{125}$I]UDR-labelled W319 C6 cultured cells to immunized and non-immunized hosts. (●) Non-immunized controls; (x) Hosts immunized with irradiated cultured or mouse-passaged cells, or viable cultured cells (results pooled). Vertical bars and numbers as in **Figure 1.**

**Figure 4** Disappearance of label (measured by external counting) after injection of [$^{125}$I]-UDR-labelled mouse-passaged cells to immunized and non-immunized hosts. (●) Non-immunized controls; (×) Immunized with irradiated mouse-passaged cells. Mice immunized with irradiated cultured cells yielded a virtually identical curve; (○) Immunized with viable cultured cells; (+) Immunized with viable mouse-passaged cells. Vertical bars and numbers as in **Figure 1.** In mice immunized with freeze-thawed cultured or mouse-passaged cells (not plotted) the pattern of loss of label was the same as in the control.

### Discussion

Our data, together with those of Jamasbi *et al.* cited below, point to the need for caution in interpreting studies with cultured human tumour lines, where there is obviously no possibility of comparison with lines passaged in the species of origin. The observed marked difference in tumorigenicity between mouse-passaged and cultured lines of murine tumours is important also because of its wider biological significance. It cannot be dismissed as an artifact due to residual FCS on the cultured cells or the loss of particular cell-surface molecules caused by exposure to trypsin during the process of cell harvesting because, as we have shown, mouse-passaged lines that are returned to culture for 48 h retain their original high tumorigenicity.

A difference in tumorigenicity between cells taken directly from a mouse and long-term cultured cells, similar to that which we have found with our fibrosarcoma lines, was observed by Jamasbi and his colleagues in experiments with chemically-induced murine squamous cell carcinomas (Jamasbi & Nettesheim, 1977, 1979). The tumours were reported to be initially highly tumorigenic and weakly immunogenic, but to become less tumorigenic and strongly immunogenic after prolonged tissue culture, and the difference in tumorigenicity was attributed to the difference in immunogencity. In many of these experiments the mice received an immunizing injection of viable cells followed by amputation of the tumour-bearing limb and rechallenge. These are difficult to interpret because metastases frequently developed from the initial inoculum and it seems likely that they influenced the response to challenge, but other experiments, referred to by Jamasbi *et al.* but not described in detail, appear to lend stronger support to their conclusions. So far as murine fibrosarcomas are concerned, however, the striking difference in tumorigenicity between mouse-passaged and cultured cell lines cannot be attributed to a difference in immunogenicity. All the cloned lines we have tested possess strong TATA, and with the two clones studied in detail we have found no significant difference in immunogenicity between the corresponding mouse-passaged and cultured lines, as judged both by the development of tumours in response to challenge, and by the kinetics of disappearance of label from the site of injection of cells labelled with [$^{125}$I]UDR in mice immunized by a previous injection of irradiated tumour cells.

The growth *in vivo* of a transplanted strongly immunogenic tumour implies that it has somehow escaped from T cell-mediated surveillance, and this in turn implies either that the inoculum contained T cell resistant tumour cells or that resistance developed sufficiently quickly for the tumour to survive despite the reaction it evoked. Our data show that the capacity to escape may be lost in the course of tissue culture but may be regained *in vivo* if the tumour is transplanted to a host which, on account of T cell or other deficiency, is unable to destroy all the potentially tumorigenic cells sufficiently quickly to prevent this from happening.

We suggested previously that the capacity of our tumours to grow in normal mice is lost during culture because, owing to lack of selective pressure, a population of cells emerges which is susceptible to destruction by both NK and/or NC cells and T cells, and that, during passage in mice deficient in T but not NK cells, these are replaced by cells which are NK- or NC-resistant, possibly owing to a surface change which makes them unrecognisable as appropriate targets, but remain sensitive to T cells. On re-transplantation to a normal mouse these passaged tumour cells then become established sufficiently quickly for further changes to occur so that the developing tumour is able to resist the T-cell mediated reaction it evokes.

In the light of the reported absence of significant NK activity in newborn mice and our observation that cultured lines passaged in newborns subsequently grew readily in normal adult mice, this hypothesis seems unlikely to be correct so far as

NK cells are concerned, though pending further evidence it cannot be excluded with certainty. So far as NC cells are concerned there are at present no grounds for rejecting the hypothesis, but there are two other possibilities which merit consideration.

Firstly, during passage in a susceptible host, the tumour cells might acquire a protective cell-surface molecule that interferes, possibly in a non-specific way, with the efferent, though not the afferent, arm of the immune response. This is certainly conceivable because antigen released from cells could immunize whereas only antigen on the cell surface provides a target for either cell-mediated or humoral cytotoxicity.

Secondly, during passage, the tumour cells might lose a molecule necessary for recognition by T cells. The mouse-passaged tumours used in this study clearly do not lack TATA but a class I MHC molecule would seem to be a candidate since the phenomenon of MHC restriction (Zinkernagel & Doherty, 1975) raises the possibility that loss of an MHC molecule required for dual recognition may provide an escape mechanism for immunogenic tumours (Woodruff, 1980).

Experiments are in progress to try to distinguish between these possibilities and to characterize the molecule or molecules concerned.

We thank Mrs E. Clark for skilled technical assistance; Dr H.T. Law, Department of Medical Physics, Western General Hospital, for access to the $^{60}$Co irradiation source; Dr C.R. Coid and Dr S. Rastan of the MRC Clinical Research Centre for supplying us with CBA nu/nu mice; Dr J. Hannan, Department of Medical Physics for lending us the scintillation detector and scaler, and Mr C. Ferrington for setting up this equipment. We thank also Prof. H.J. Evans for the privilege of working in his Unit, and the Medical Research Council, UK, for a Project Grant.

### References

HERBERMAN, R.B., NUNN, M.F. & LAVRIN, D.H. (1975). Natural cytotoxic reactivity of mouse lymphoid cells against syngeneic and allogeneic tumours. I. Distribution of reactivity and specificity. *Int. J. Cancer,* **16,** 216.

JAMASBI, R.J. & NETTESHEIM, P. (1977). Increase in immunogenicity of a pulmonary squamous-cell carcinoma, propagated *in vitro. Int. J. Cancer,* **20,** 817.

JAMASBI, R.J. & NETTESHEIM, P. (1979). Increase in immunogenicity with concomitant loss of tumorigenicity of respiratory tract carcinomas during *in vitro* culture. *Cancer Res.,* **39,** 2466.

KIESSLING, R., KLEIN, E., PROSS, H. & WIGZELL, H. (1975). Natural killer cells in the mouse. II. Cytotoxic cells with specificity for mouse Moloney leukaemia cells. Characteristics of the killer cell. *Eur. J. Immunol.,* **5,** 117.

KIESSLING, R. & WIGZELL, H. (1979). An analysis of the murine NK cell as to structure, function and biological relevance. *Immunol. Rev.,* **44,** 165.

LATTIME, E.C., PECORARO, G.A., CUTTITO, M.J. & STUTMAN, O. (1983). Murine non-lymphoid tumours are lysed by a combination of NK and NC cells. *Int. J. Cancer,* **32,** 523.

STUTMAN, O., PAIGE, C.J. & FEO FIGARELLA, E. (1978). Natural cytotoxic cells against solid tumours in mice. I. Strain and age distribution and target cell susceptibility. *J. Immunol.,* **121,** 1819.

WOODRUFF, M.F.A. (1980). *The Interaction of Cancer and Host: Its Therapeutic Significance.* New York: Grume and Stratton, p. 135.

WOODRUFF, M.F.A., ANSELL, J.D., FORBES, G.M., GORDON, J.C., BURTON, D.I. & MICKLEM, H.S. (1982*a*). Clonal interaction in tumours. *Nature,* **299,** 822.

WOODRUFF, M.F.A., ANSELL, J.D., HODSON, B.A. & MICKLEM, H.S. (1984*a*). Specificity of tumour associated transplantation antigens (TATA) of different clones from the same tumour. *Br. J. Cancer,* **49,** 5.

WOODRUFF, M.F.A., FORBES, G. & GORDON, J. (1982*b*). Immunogenicity, macrophage sensitivity, and therapeutic response to C. *parvum* of fibrosarcomas induced in C. *parvum*-treated and untreated mice. *Cancer Immunol. Immunoth.,* **12,** 255.

WOODRUFF, M.F.A., HODSON, B.A. & ANSELL, J.A. (1984*b*). Transplantability of tumour cell clones before and after passage in immunodeficient hosts. *Br. J. Cancer,* **49,** 396.

ZINKERNAGEL, R.M. & DOHERTY, P.C. (1975). H-2 compatibility requirement for T cell mediated lysis of target cells infected with lymphocytic choriomeningitis virus. Different cytotoxic T cell specificities are associated with structures coded for in H-2K or H-2D. *J. Exp. Med.,* **141,** 1427.